# The cytology of micropapillary variant of colloid carcinoma of breast: A report of two cases

**DOI:** 10.4103/0970-9371.70751

**Published:** 2010-04

**Authors:** Jayashree Krishnamurthy, Divya Kota Nagappa

**Affiliations:** Department of Pathology, Medical College (VIMS), Bellary - 583 104, Karnataka India

**Keywords:** Colloid carcinoma, fine-needle aspiration cytology, micropapillary variant, pure mucinous carcinoma

## Abstract

Colloid carcinoma (pure mucinous carcinoma) is an uncommon variant of breast carcinoma with distinctive cytological and histological features. These tumors are characterised by islands of tumor cells floating in a sea of abundant extracellular mucin. We present the cytology of two cases of colloid carcinoma occurring in 80-year-old and 45-year-old females. The fine-needle aspiration cytology helps to subcategorize the tumor type, thereby enhancing the knowledge about the distinctive cytological features of special and uncommon variants of breast carcinoma, their course and prognosis. A distinctive micropapillary variant of pure mucinous carcinoma which is rarely described, is represented in one of the cases. Also we report, here, colloid carcinoma in a female of reproductive age, a relatively uncommon occurrence.

## Introduction

Colloid carcinoma (pure mucinous carcinoma) is an unusual invasive type of breast carcinoma accounting for 1–6% of all breast carcinomas.[1] The uniqueness of colloid carcinoma of the breast is the presence of islands of tumor cells floating in abundant extracellular mucin.

The cytological features of two cases of colloid carcinoma occurring in an 80-year-old female and a 45-year-old female are presented here.

## Case Reports

### Case 1

An 80-year-old postmenopausal female presented with a slowly growing painless swelling in the right-sided breast of two years’ duration. Local examination revealed a well circumscribed, mobile, soft-to-firm swelling measuring 3×2 cm. in the upper outer quadrant of the right breast. The nipple and areola were normal and the swelling was not attached to the chest wall. The general physical and systemic examinations were normal. There was no axillary lymph node enlargement.

### Case 2

A 45-year-old female presented with a left-sided breast swelling of six months’ duration. The patient had not attained menopause. Local examination revealed a well circumscribed, firm swelling measuring 6×5 cm. It was nontender with restricted mobility, in the outer lower quadrant of the left breast. The nipple and areola were normal. The general physical and systemic examinations were normal. Axillary lymph nodes were not palpable.

The routine investigations were within normal limits in both the cases.

**Figure 1:**
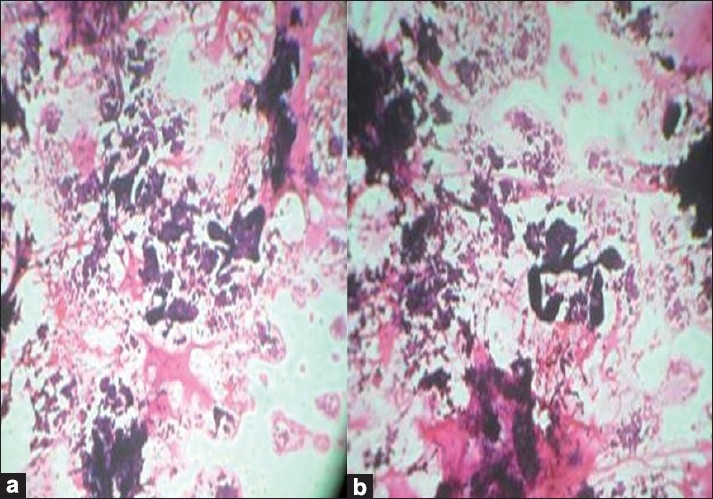
(a) Microphotograph of smear showing tumor cells floating in a sea of mucin (H and E × 100), (b) Microphotographs of smear showing micropapillae in a mucoid background (H and E×100)

**Figure 2:**
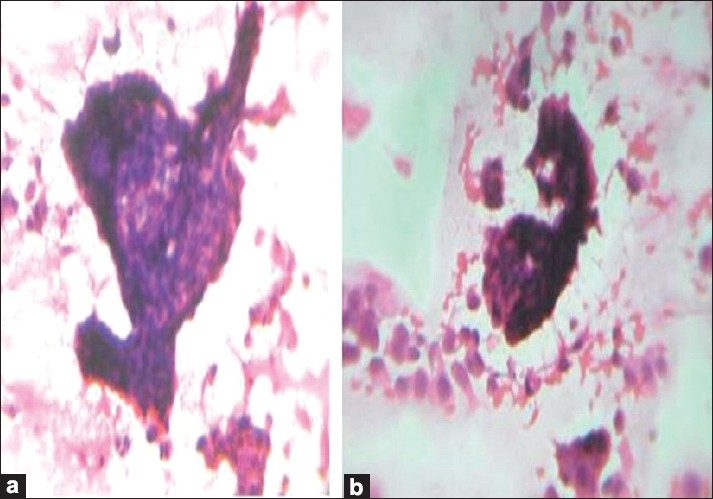
(a and b): Microphotograph of smears of micropapillae of tumor cells without fibrovascular core in mucoid background (H and E, ×400)

**Figure 3:**
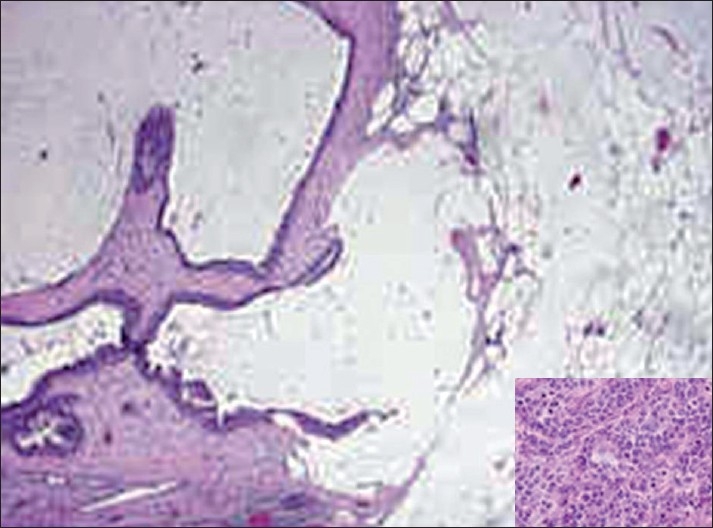
Microphotograph of section showing mucinous carcinoma (H and E, ×100) with micropapillary architecture in the inset (H and E, ×400).

Fine-needle aspiration cytology (FNAC) of the breast swelling was done in both the cases using a 24-gauge needle and 10-ml syringe to obtain a thick white aspirate. On spreading, the aspirate was quite glary, hinting at a high mucin content. The smears were stained with hematoxylin-eosin (H and E) and May-Grünwald-Giemsa (MGG) stains. The smears were highly cellular. The epithelial cells were distributed as loosely cohesive groups, flat sheets and single cells bathed in abundant mucin of variable density. The mucin was homogenous and extracellular [Figure 1]. The epithelial cells were monomorphic, round to oval with eccentric or central round or oval vesicular nuclei [Figure 1]. The nuclei showed smooth nuclear outlines, were bland, with possibly granular chromatin and inconspicuous nucleoli. Myxovascular fragments which correspond to thin endothelial vessels lying in the mucin were seen in both the cases [Figure 1]. Case 2 showed many micropapillae of mildly pleomorphic cells in a mucoid background. True tumor papillae with fibrovascular cores were not present [Figure 2]. A cytological diagnosis of colloid carcinoma or pure mucinous carcinoma of breast was suggested in case one; and colloid carcinoma, micropapillary variant, in case two. The diagnosis was confirmed by a histopathological examination in both the cases [Figure 3].

## Discussion

Colloid carcinoma or pure mucinous carcinoma is typically a tumor of older, postmenopausal women usually at an age of 60 years or more.[1] In the present report, one of the patients was in the reproductive age group. In the study by Jayaram *et al*.,[2] mucinous carcinomas occurred in a wide group ranging from 38 to 90 years. It tends to be slow growing. On examination, these lesions tend to be well defined with smooth outline, hard and mobile and hence may be mistaken clinically for a fibroadenoma or cyst.[3] Mucinous carcinoma is associated with a very low incidence [2-4%] of nodal metastases.[4] It carries an excellent short-term prognosis particularly when the tumor measures less then 3cm in diameter. The deaths from this tumor can occur many years after therapy indicating the need for a long-term follow-up.

The cytological descriptions of mucinous carcinoma were described by Fanning *et al*.[5] and are identical to the cytological features noted here for both the cases. The myxovascular fragments seen in both the cases probably correspond to the thin endothelial vessels lying in the mucin and were seen in 50% of mucinous carcinoma cases. The uncommon micropapillary architecture of mammary mucinous carcinoma seen in the present report has been described in less than 1% of cases, in a review by Wai-Kuen.[6] The micropapillae are not true papillae with fibrovascular core and may represent the mucinous counterpart of invasive micropapillary carcinoma. About a fourth to nearly half of mucinous carcinoma show features consistent with endocrine differentiation raising a possibility of a link between mucinous carcinoma and carcinoid of breast.[7]

The cytological differential diagnosies of mucinous carcinoma include mucocele-like lesion, mixed mucinous-infiltrating carcinoma, myxoid fibroadenoma, secretory carcinoma and mucinous cystadenocarcinoma.[8]

Mucocele-like lesions show abundant extracellular mucin, few cohesive flat sheets of epithelial cells with uniform round nuclei, indiscernible nucleoli and fine chromatin.[5]

Myxoid fibroadenoma presents in the younger age group, and has cytological features of branching monolayered sheets of tightly cohesive benign ductal epithelial cells, bipolar naked nuclei and stromal fragments.[2]

Mixed mucinous-infiltrating carcinoma shows cellular pleomorphism, scanty amount of mucin and necrosis.[9]

Secretory carcinoma of the breast presents in the younger age group and has cytological features of intracellular and extracellular mucin, many signet ring cells, vacuolated cells and mucoglobular structures resembling bunch of grapes.[10]

Mucinous cystadenocarcinoma has columnar cells with intracytoplasmic mucin giving the appearance of signet ring cells.[2]

Mucocele-like lesions encompass a spectrum of pathological lesions including atypical ductal hyperplasia, intraductal carcinoma and invasive mucinous carcinoma.[11] Therefore cytological evaluation of breast lesions containing abundant extracellular mucin should be done carefully to diminish the likelihood of over-diagnosis and under-diagnosis.

The monomorphic features of pure mucinous carcinoma may give a false impression of benignancy. Hence all breast lesions containing abundant mucin should be excised for histological examination because aspiration cytology of these lesions may be misleading.

In conclusion, knowledge of distinctive cytomorphological appearance of colloid carcinoma would enable correct identification of these lesions as malignant and prompt treatment that could further enhance the survival of these prognostically good breast cancers.
